# Stimuli-Free Transcuticular Delivery of Zn Microelement Using Biopolymeric Nanovehicles: Experimental, Theoretical, and *In Planta* Studies

**DOI:** 10.1021/acsnano.1c06161

**Published:** 2021-11-24

**Authors:** Yael Cohen, Hagai Yasuor, Dmitry Tworowski, Elazar Fallik, Elena Poverenov

**Affiliations:** † Agro-Nanotechnology and Advanced Materials Center, Institute of Postharvest and Food Sciences, Agriculture Research Organization, The Volcani Institute, Rishon LeZion 7505101, Israel; ‡ Institute of Biochemistry, Food Science and Nutrition, Faculty of Agriculture, Food and Environment, The Hebrew University of Jerusalem, Rehovot 76100, Israel; § Department of Vegetables and Field Crops, Agriculture Research Organization, Gilat Center, M.P. Negev 85280, Israel; ∥ Department of Structural Biology, Weizmann Institute of Science, 76100 Rehovot, Israel

**Keywords:** carboxymethyl cellulose, foliar nutrition, polysaccharide nanovehicles, microelements, zinc, delivery, 3-D structural modeling

## Abstract

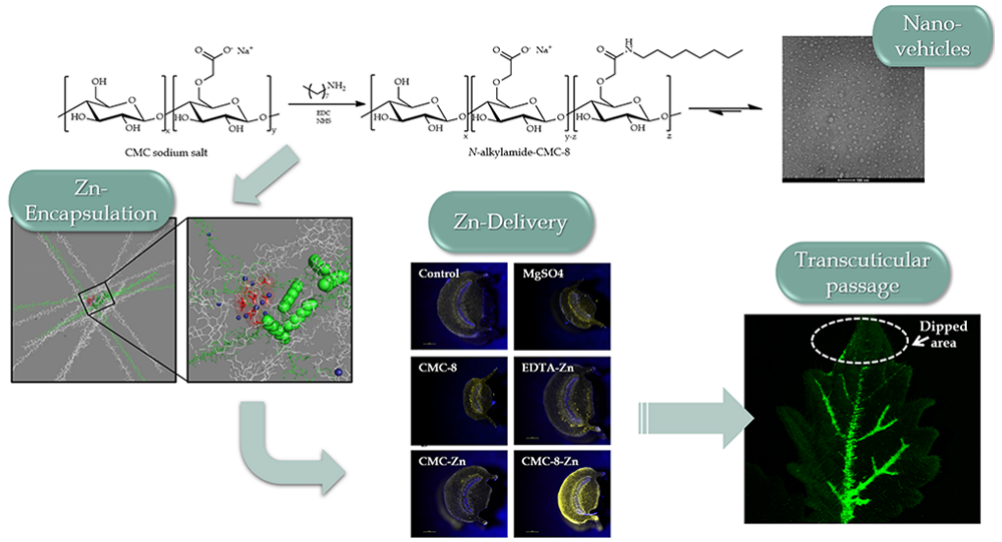

This paper reports one-step synthesis of polysaccharide-based nanovehicles, capable of transporting ionic zinc *via* plant cuticle without auxiliary stimulation. Delivery of highly hydrophilic nutritive microelements *via* the hydrophobic cuticle of plant foliage is one of the major challenges in modern agriculture. In traditional nutrition *via* roots, up to 80% of microelements permeate to soil and get wasted; therefore, foliar treatment is an environmentally and economically preferable alternative. Carboxymethyl cellulose (CMC) was modified to amphiphilic *N*-octylamide-derivative (CMC-8), which spontaneously self-assemble to nanovehicles. It was found that hydrophobic substituents endow a biopolymer with unexpected affinity toward a hydrophilic payload. CMC-8 nanovehicles effectively encapsulated ionic zinc (ZnSO_4_) and delivered it upon foliar application to pepper (*Capsicum annuum*) and tomato (*Solanum lycopersicum*) plants. Zinc uptake and translocation in plants were monitored by SEM-EDS and fluorescence microscopic methods. *In planta* monitoring of the carrier was done by labeling nanovehicles with fluorescent carbon dots. Three-dimensional (3-D) structural modeling and conformational dynamics explained the CMC-8 self-assembly mechanism and zinc coordination phenomenon upon introduction of hydrophobic substituents.

In modern plant cultivation, there is a heavy shortage of nutritional microelements from natural sources, and their external supply has been practiced worldwide.^1,2^ Plant nutrition is routinely done *via* root systems using irrigation with nutrient-rich water (fertigation) or by adding solid fertilizers to soil. However, the root-based nutrition approach suffers from serious drawbacks, such as (a) low efficacy and economical invalidity, because most of the supplied bioactive compound does not reach the plant but is washed away with irrigation or rainwater ; (b) undesired environmental impact and pollution of ground water; and (c) lack of control of the amount of active agents intake by plants.^3^ In the foliar nutrition, microelements are directly applied on plant leaves,^4^ an economically and environmentally favorable alternative^5^ that also benefits from higher efficacy.^6,7^ The main problem associated with foliar application is the obstruction of hydrophilic nutritional microelement penetration by lipophilic cuticles on plant leaves.^8,9^ Microelements can enter the leaves through stomata but not as a mass flow.^9−12^ Numerous studies have been dedicated to understanding foliar uptake of ionic solutes by investigating the influence of humidity, temperature, microelement ion size, and surfactants on this process.^12−14^ Zinc microelement is necessary for normal plant development. Its deficiency is associated with the disruption of enzymatic processes, leading to inhibition of photosynthesis, increasing membrane leakiness, and other harmful processes.^15,16^

With significant progress in materials science and nanotechnology, advanced delivery systems have been suggested for agricultural applications in general and for foliar delivery in particular.^17−19^ The advanced nanomaterials that have been reported to pass through plant cuticle are nanostructured liquid crystalline particles,^20^ coated gold nanoparticles,^21^ aerosol nanoparticles,^22^*etc*. Specifically, for transcuticular delivery of microelements, biofunctional microgels,^23^ nanoparticle–liposome composites,^24^ and metal–organic frameworks (MOFs)^25^ were reported. In the design of all these advanced nanomaterials, the nature of the carrier material plays a crucial role in their success. Among the different carriers for plant application, polysaccharides seem to be very promising candidates, as they are green, biocompatible, and inexpensive. Polysaccharides have been successfully applied for biomedical, food, and cosmetics applications.^26−28^ In plants, polysaccharide-based systems were reported to perform foliar delivery of hydrophobic cargo, such as pesticides and hormones.^29−32^ Delivery of microelements remains quite naive for polysaccharide-based delivery systems. To the best of our knowledge, only one work, where the foliar delivery of zinc was done by nanoparticles that were formed by reacting chitosan with tripolyphosphate cross-linker, has been reported in this regard.^33^

The modified amphiphilic polysaccharides that are capable of spontaneous self-assembling are regarded to be the next-generation delivery materials as they have demonstrated excellent performance in drug delivery.^34−36^ Recently, it was reported that self-assembling modified polysaccharides are capable of successfully transporting molecular cargo *via* hydrophobic biological barriers. For instance, they showed efficient transdermal delivery of hydrophilic molecules.^37−40^ The successful transdermal delivery encouraged us to examine self-assembled polysaccharides for foliar delivery of nutrients in plants. However, this objective is very challenging, as it involves encapsulation and transportation of extremely hydrophilic ionic microelements across the highly hydrophobic cuticle of the leaves.^41^ In addition, plants can be pH-sensitive; therefore, using polysaccharides that do not require acidic or basic pH for their dissolving are desired.

In this paper, we aimed to develop a polysaccharide-based system, capable of encapsulating a hydrophilic plant nutrient, ionic zinc, and deliver it through the hydrophobic cuticular leaves. Carboxymethyl cellulose (CMC) was modified to form amphiphilic *N*-octylamide-derivative (CMC-8) vehicles that have effectively encapsulated zinc sulfate (ZnSO_4_) and delivered it *via* foliar application to pepper (*Capsicum annuum*) and tomato (*Solanum lycopersicum*) plants. Fluorescent carbon dots were synthesized and covalently attached to CMC-8 vehicles for *in planta* monitoring of their uptake and translocation by the plant leaves. Molecular dynamic calculations explained the self-assembly mechanism of CMC-8 and the observed phenomenon, where introduction of hydrophobic substituents provided the modified polysaccharide with an ability to coordinate and transport the highly hydrophilic payload.

## Results and Discussion

**Figure 1:**
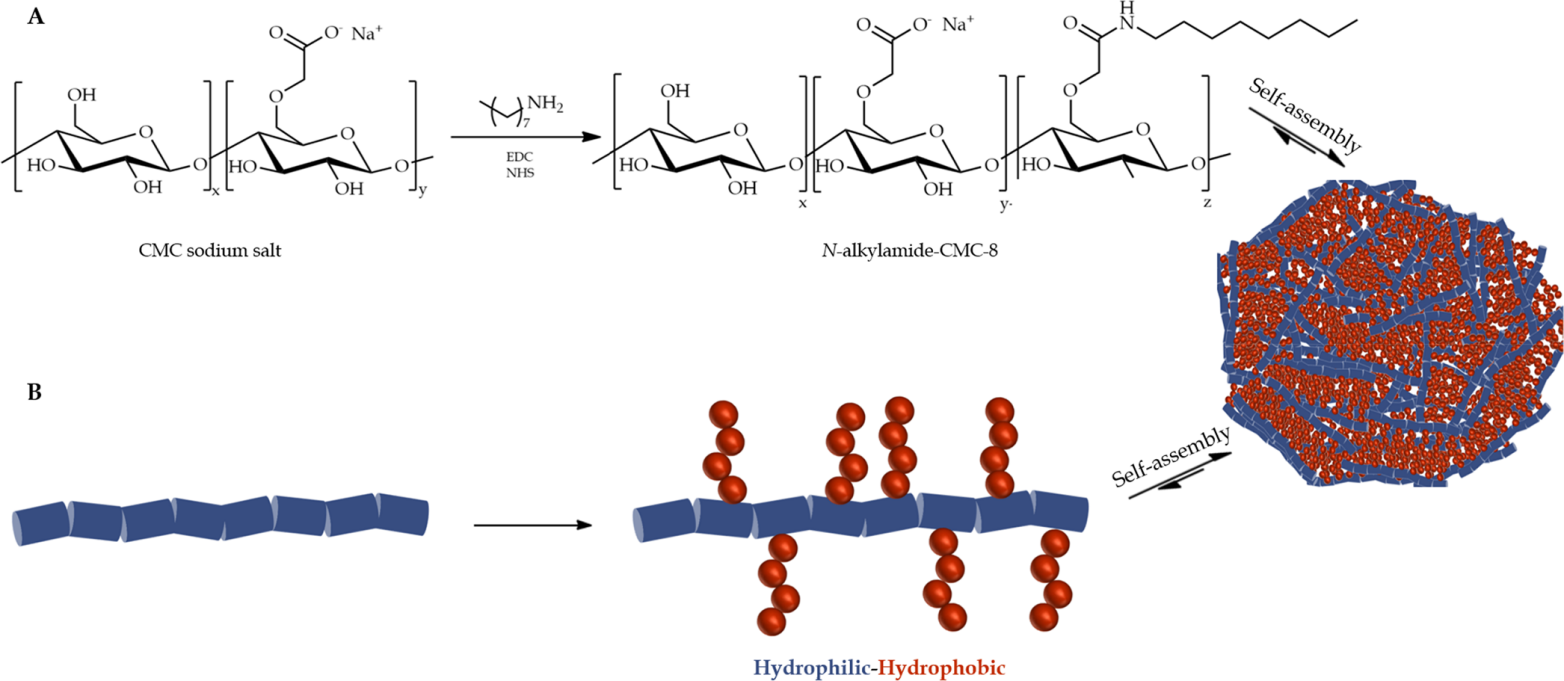
(A) Synthesis of *N*-octyl-amide polysaccharide CMC-8 and (B) schematic representation of the formation of self-assembled aggregate of CMC-8.

*N*-Octylamide-substituted CMC polysaccharide, termed CMC-8, was synthesized *via* a one-pot reaction between carboxymethyl cellulose (CMC) and octyl amine, utilizing *N*-hydroxysuccinimide as a catalyst and 1-ethyl-3-(3-(dimethylamino)propyl)carbodiimide hydrochloride as a coupling agent (Figure 1). Octyl substituents provided the polysaccharide with amphiphilicity, allowing spontaneous self-assemble to form nanosized aggregates above the “critical aggregation concentration”, found to be 0.62 ± 0.12 mg/mL. Notably, the original CMC did not show any aggregation and self-assembly ability. Transmission electron microscopy (TEM) confirmed the formation of CMC-8 nanostructures (Figure 7). A distinctive along-chain modification mode provided the resulting CMC-8 with a dynamic structure enabling encapsulation of not only hydrophobic but also hydrophilic and even ionic guests. Unlike classic micelles, which possess a hydrophobic core and hydrophilic shell, the CMC-8 aggregates have simultaneous hydrophilic and hydrophobic areas and were further confirmed in this work by molecular modeling and conformational analysis.

**Figure 2:**
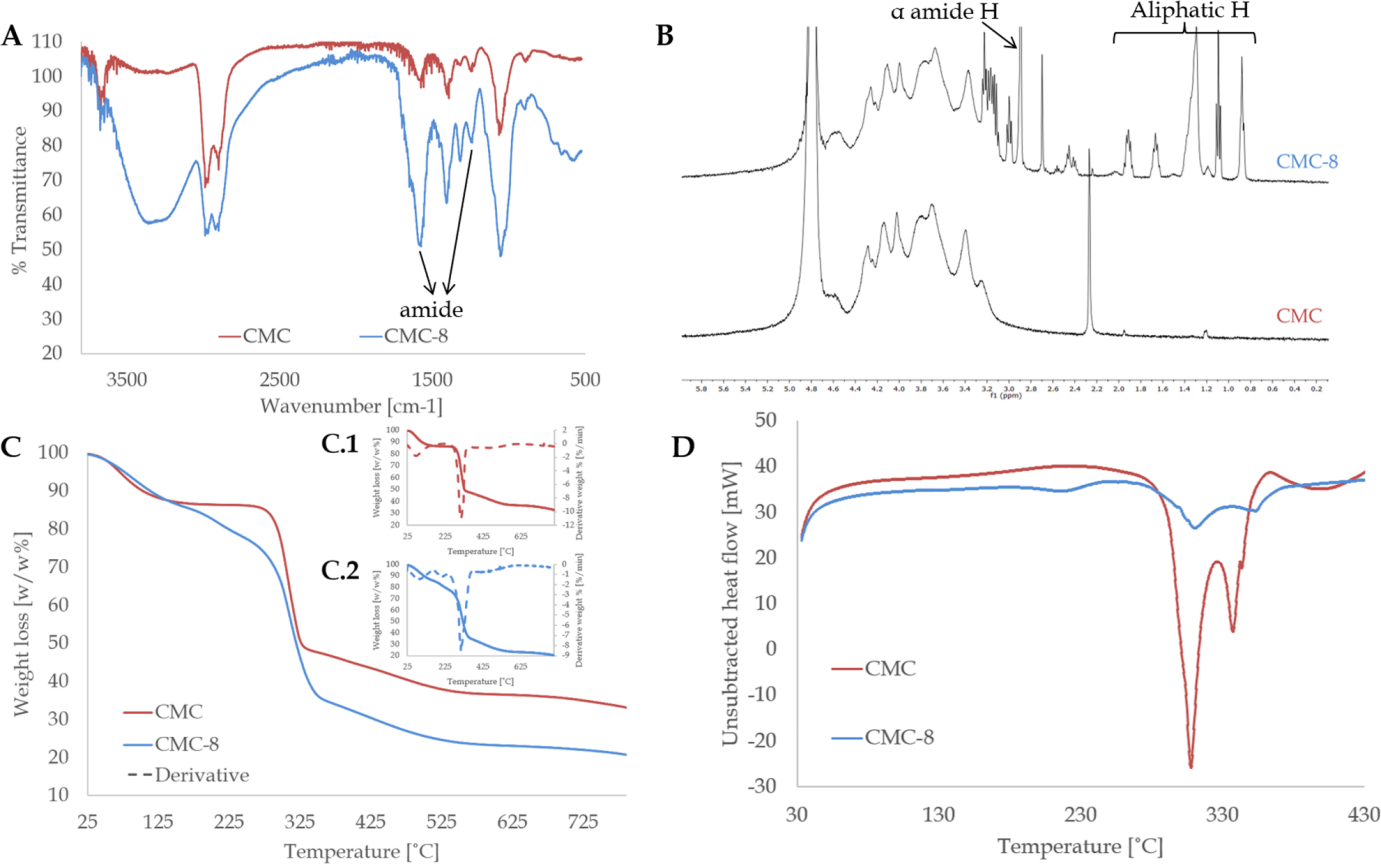
(A) ATR-FTIR spectra, (B) 400 MHz ^1^H NMR spectra, (C) TGA diagrams with the derivative curves in the inset (C.1 and C.2) and (D) DSC diagrams of the original CMC and CMC-8.

The ATR-FTIR and ^1^H NMR spectroscopy confirmed the formation and isolation of CMC-8 product with no trace of the reactants (Figure 2A, B). ATR-FTIR spectrum showed an aliphatic peak at 1317 cm^–1^, related to the coupled octyl chain (alkane, C–H, bending); the amide carbonyl and N–H bending frequencies and C–N stretch frequencies appeared at 1580 cm^–1^ 1249 cm^–1^, respectively. ^1^H NMR scans have displayed the aliphatic protons of the coupled alkyl chain at 0.86–1.91 ppm and α amide protons at 2.9 ppm. The degree of CMC-8 substitution was calculated using total organic carbon method and was found to be 22%.

Thermal gravimetric analysis (TGA) and differential scanning calorimetry (DSC) studies of CMC-8 were performed and compared to that of the original CMC (Figure 2 C, D). The modification did not lead to drastic changes in the thermal profile of the modified polymer. In TGA, CMC displayed a relatively linear pattern, while CMC-8 displayed a declining pattern until the large pyrolytic events occurred at 290.9 and 290.4 °C (according to their derivative curves) with weight loss of 47.9% and 52.9% and overall weight loss of 66.6% and 79.0% for CMC and CMC-8, respectively. CMC-8 displayed an additional event at 190.3 °C, which might be related to the breaking down of the amide bonds between the aliphatic chains and CMC.^42^ In DSC studies, CMC and CMC-8 also showed similar diagrams with the decomposition temperatures above 280 °C, indicating thermal resistance. Although octyl amine substitution barely affected the polymer’s thermal stability, the two polymers displayed differences in their decomposition patterns, evidencing modification.

The CMC-8 and CMC polymers were examined by gel permeation chromatography, utilizing dextran as a calibrating standard to determine the number-average molecular weight (*M*_n_) as a function of retention time, resulting in *M*_n_ of 1277 kDa for CMC-8 and 154 kDa for CMC. It is important to note that spatial structure significantly influences polymer’s retention time. Because the original CMC has no assembling abilities and CMC-8 is a self-assembled aggregate, the received *M*_n_ values illustrate a difference between the two polymers but cannot be taken for precise evaluation of molecular weight.^43^

### Computational Modeling Study

**Figure 3:**
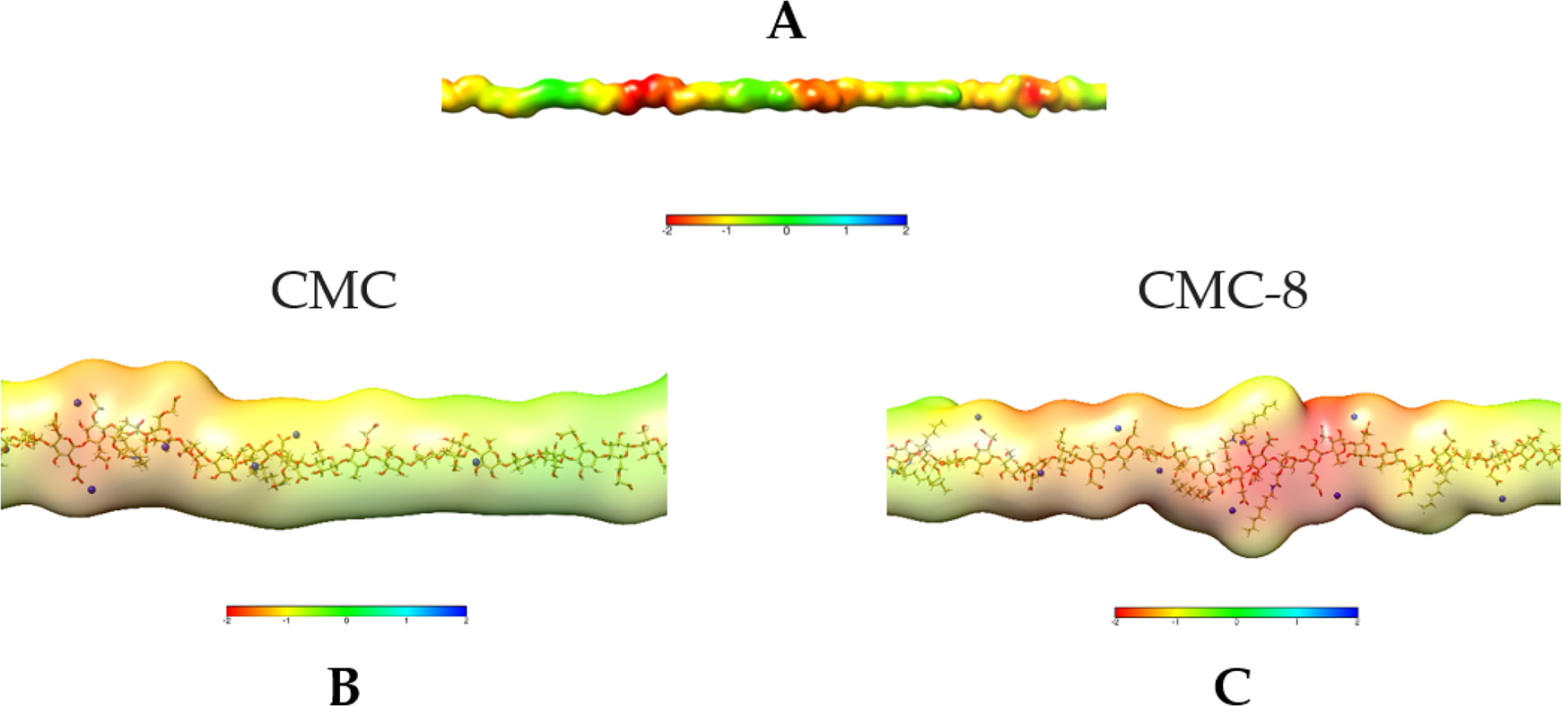
Color maps represent the 3D distribution of the electrostatic potential along CMC (A and B) and CMC-8 (C) chain ranging from the negative (red and yellow) to the neutral and positive (green and blue) values. Positive Zn^2+^ ions shown as small dark-blue spheres tend to diffuse into the negative electrostatic potential areas. Structures and surface maps were visualized in Chimera.^44^

Three-dimensional structural modeling, continuum electrostatics calculations, and analysis of polymer chain conformational dynamics were performed to get insight into the self-assembly mechanism of CMC-8 and to understand how the introduction of hydrophobic substituents has led to the enhancement in affinity to highly hydrophilic ionic zinc. The molecular composition of the studied polymers is extremely complex and was found to contain 27 different types of monomers (Figures S1–S3). Based on these monomers, a library of random 100 and 1000 length polymer chains was generated (as described in Experimental Section). It was found that negative electrostatic potential is nonuniformly distributed along the polymer chain but is concentrated near 2,3,6-tri-O-(carboxymethyl)glucose monomers possessing the highest negative total charge of −3*e*. Theoretically, there are ∼29–30 such negative potential sites per one 1000 monomer length CMC molecule and ∼22 sites per one 1000 monomer length CMC-8 molecule (Figure 3). It is reasonable to assume these negatively charged sites are favorable for Zn^2+^ binding.

**Figure 4:**
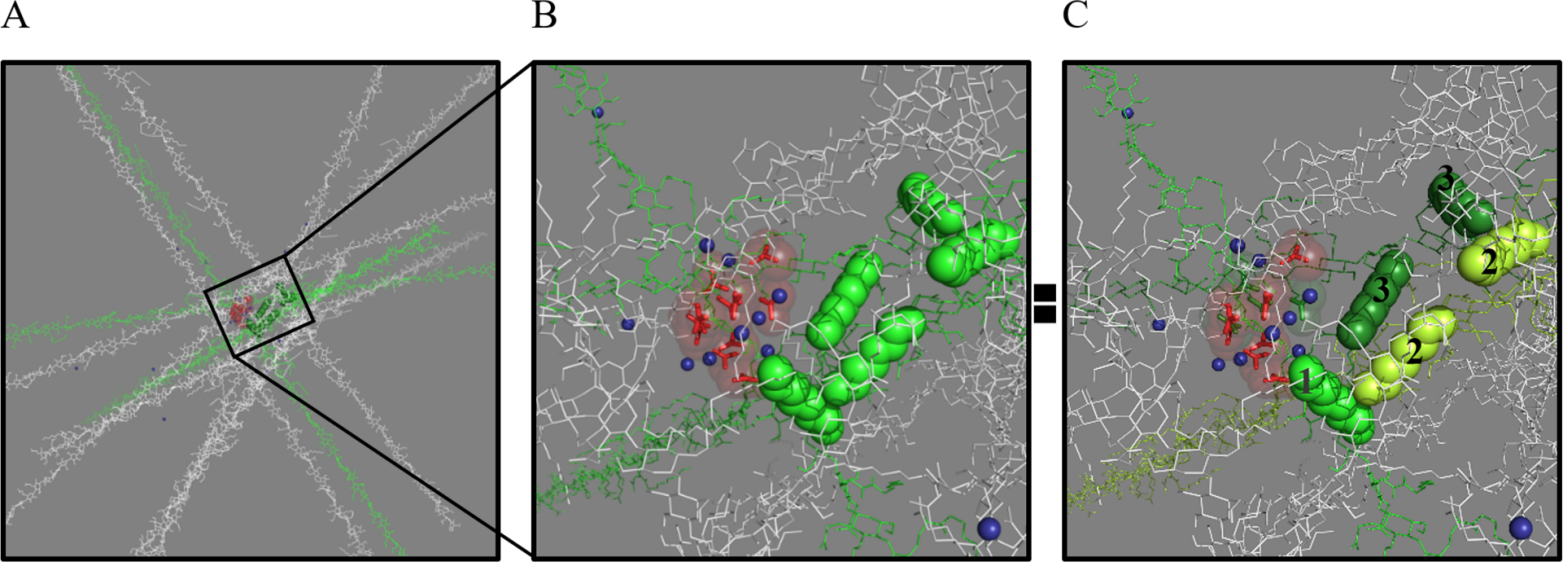
CMC-8 molecules. (A) Three polymer molecules (green sticks) form an alkyl-8 hydrophobic cluster (green spheres). For clarity, only 10 polymer molecules (white and green sticks) are shown. (B) The red “cloud” represents strong negative electrostatic potential generated by the cluster of 9 COO^–^ groups (red sticks). Positive Zn^2+^ ions (small dark-blue spheres) tend to move/diffuse into the areas of the negative electrostatic potential. The alkyl hydrophobic cluster (green spheres) is created by 5 alkyl groups. (C) Same as panel B except for the green color change. The binding stoichiometry in atomic detail: The alkyl-8 hydrophobic cluster is created by 5 alkyl groups from three polymer molecules 1 (green), 2 (lemon), and 3 (forest); each alkyl from the molecule 2 interacts with one alkyl of the molecule 3, and one of the “lemon” alkyl groups also binds with the “green” alkyl of the molecule 1. Figures were prepared in PyMOL.^45^

Interestingly, according to experimental data, despite the similar and even lower amount of potential Zn^2+^ binding sites, CMC-8 is a more pronounced Zn^2+^ carrier compared to the unmodified CMC. This phenomenon was further explained by conformational dynamics analysis, which revealed that hydrophobic interactions between the alkyl substituents in CMC-8 drive the self-assembly of semiflexible polymer chains and promote the formation of compact structures (Figure 4). Because of such aggregation, stronger electrostatic potential is generated by several negatively charged sites, forming the areas with strong electrostatic affinity toward Zn^2+^ ions. On the other hand, the unmodified CMC polymers do not undergo self-assembly, and the negative electrostatic potential generated by a single charged site is not sufficient for the strong binding of Zn^2+^.

The close insight into the mechanism of Zn coordination by the self-assembled CMC-8 is illustrated as follows. In CMC-8, there are approximately six to seven “hydrophobic spots” with alkyl–alkyl chain interactions that can trigger a hydrophobic patch formation and spontaneous self-assembly. For example, three polymer molecules (green sticks, Figure 4A) form an alkyl hydrophobic cluster (green spheres). In proximity to the alkyl cluster (∼10–15 Å apart), carboxy groups from different polymer chains create the Zn^2+^ binding site (red area, Figure 4A). Upon a closer look on the binding site, the red “cloud” around the red sticks represents strong negative electrostatic potential generated by the cluster of nine COO^–^ groups. Mobile Zn^2+^ cations (dark-blue spheres) tend to move toward the area on strong negative potential (Figure 4B).

More atomic details on the binding stoichiometry are highlighted in Figure 4C. Specifically, the alkyl hydrophobic cluster is created by five alkyl groups from three different polymer molecules (green-1, lemon-2, and forest-3): each alkyl from the lemon molecule interacts with one alkyl from the forest molecule. In addition, one lemon’s alkyl group interacts with green’s alkyl. The carboxyl cluster composition (red sticks) includes four and three carboxy groups from the green and lemon polymers, respectively, and three carboxyls from a nearby environment “white” molecule (Figure 4C). The electrostatic potential (red “cloud”), generated by this acidic cluster can accumulate up to 7–8 Zn^2+^ ions (dark-blue spheres). Figure 4 represents a typical pattern of interactions that drive the self-assembly of CMC-8 aggregates and their ability of Zn^2+^ binding.

Thus, although pristine CMC has numerous sites for ionic interactions between Zn^2+^ and COO^–^, these interactions are dynamic and Zn ions can easily decoordinate in an aqueous solution. On the other hand, in the self-assembled CMC-8, clouds of negative electrostatic potential are generated by numerous COO^–^ groups. In such clouds, electrostatic interactions with Zn^2+^ ions are stronger. In addition, because of trapping inside the nanovehicle that also has hydrophobic moieties, Zn^2+^ ion migration back to the bulk aqueous solution is therefore restricted/limited. Together these factors endow CMC-8 with stronger Zn^2+^ encapsulation capability in comparison to the unmodified polymer.

### Foliar Delivery of Ionic Zinc

**Figure 5:**
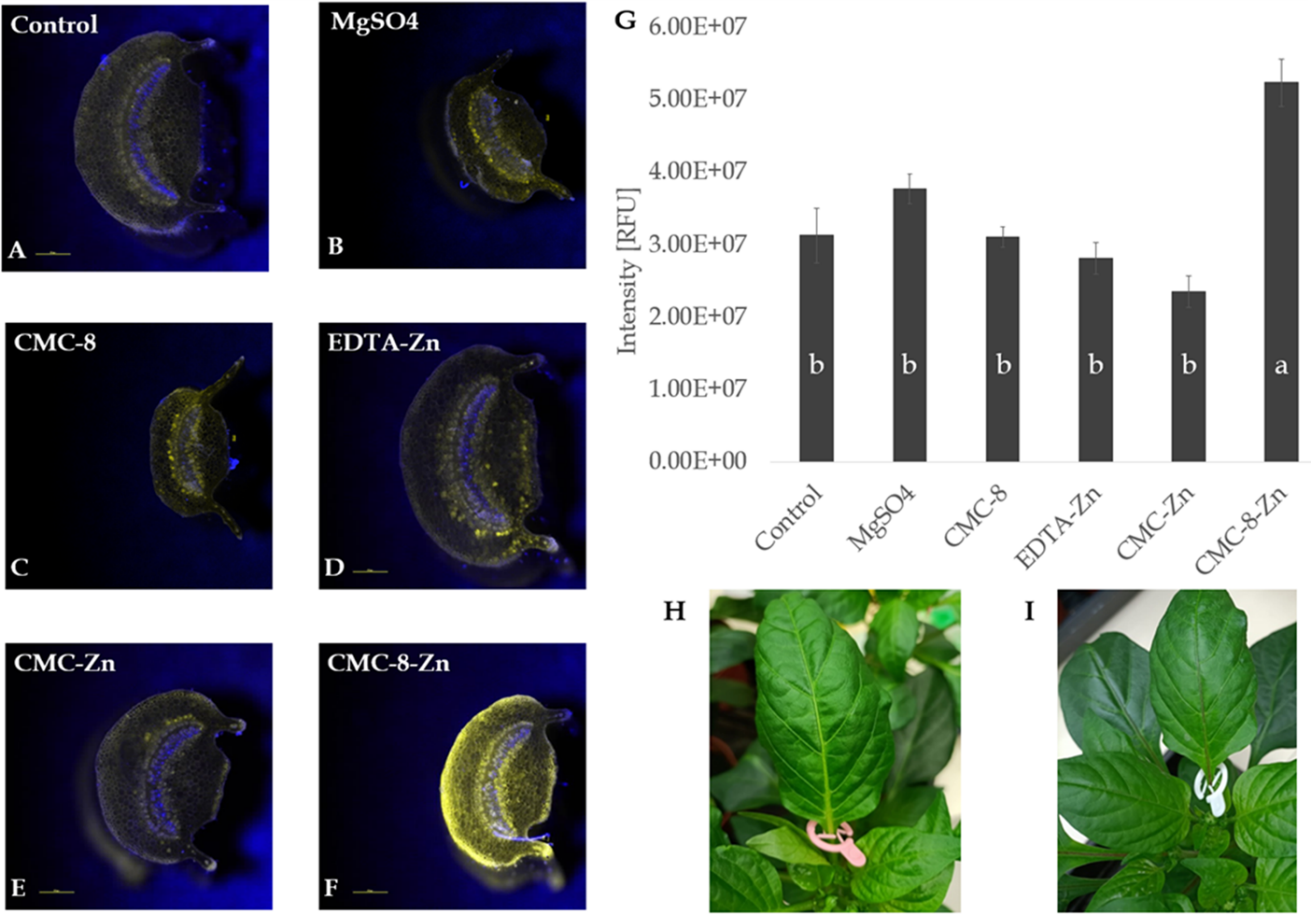
(A–F) Fluorescence stereoscope images and (G) fluorescence quantification concentration of zinc stained with zynpir-1 (10 μM for 1 h in the dark at room temp). Fluorescent images are cross sections of pepper leaf petiole down the treated area in the presence of CMC-8, EDTA, and CMC delivery systems. Zinc concentration was set to 500 ppm. All samples were tested in eight repetitions. Distilled water treatment, MgSO_4_, and CMC-8 were used as the negative controls. Values with different letters are significantly different according to Tukey HSD test at *p* ≤ 0.05. (H and I) Pepper leaf images after 24 h (H) control and (I) CMC-8 treatments.

The ability of CMC-8 to serve as nanovehicles for delivery of ionic zinc was examined. Zinc is a vital microelement for plant nutrition, and foliar delivery of zinc is extremely challenging because the highly hydrophilic nature of zinc ions obstructs their permeation *via* the hydrophobic barrier of the plant cuticle.^46^ The aqueous solution of CMC-8 loaded with ZnSO_4_ (500 ppm) was dropped on the leaf of pepper plant (*C. annuum*), and the microelement’s distribution in the leaf petiole was monitored using zinc-staining fluorescence dye (Zinpyr-1). The delivery of ionic zinc by unmodified CMC and ethylenediaminetetraacetic acid (EDTA), the traditional chelating agent used for foliar application of microelements, were also examined for comparison. Distilled water treatment, without the addition of zinc, was used as a negative control. It can be seen that CMC-8 delivery systems resulted in a significant increase of zinc concentration in qualitative and quantitative measurements, indicating their ability to enhance the foliar delivery of highly hydrophilic ionic zinc. EDTA and unmodified CMC did not result in any significant increase. CMC-8 treatment did not show any visual toxicity. The treated leaves remained green, healthy, and unharmed, as can be seen in their images that were taken 24 h after the treatment (Figure 5).

**Figure 6:**
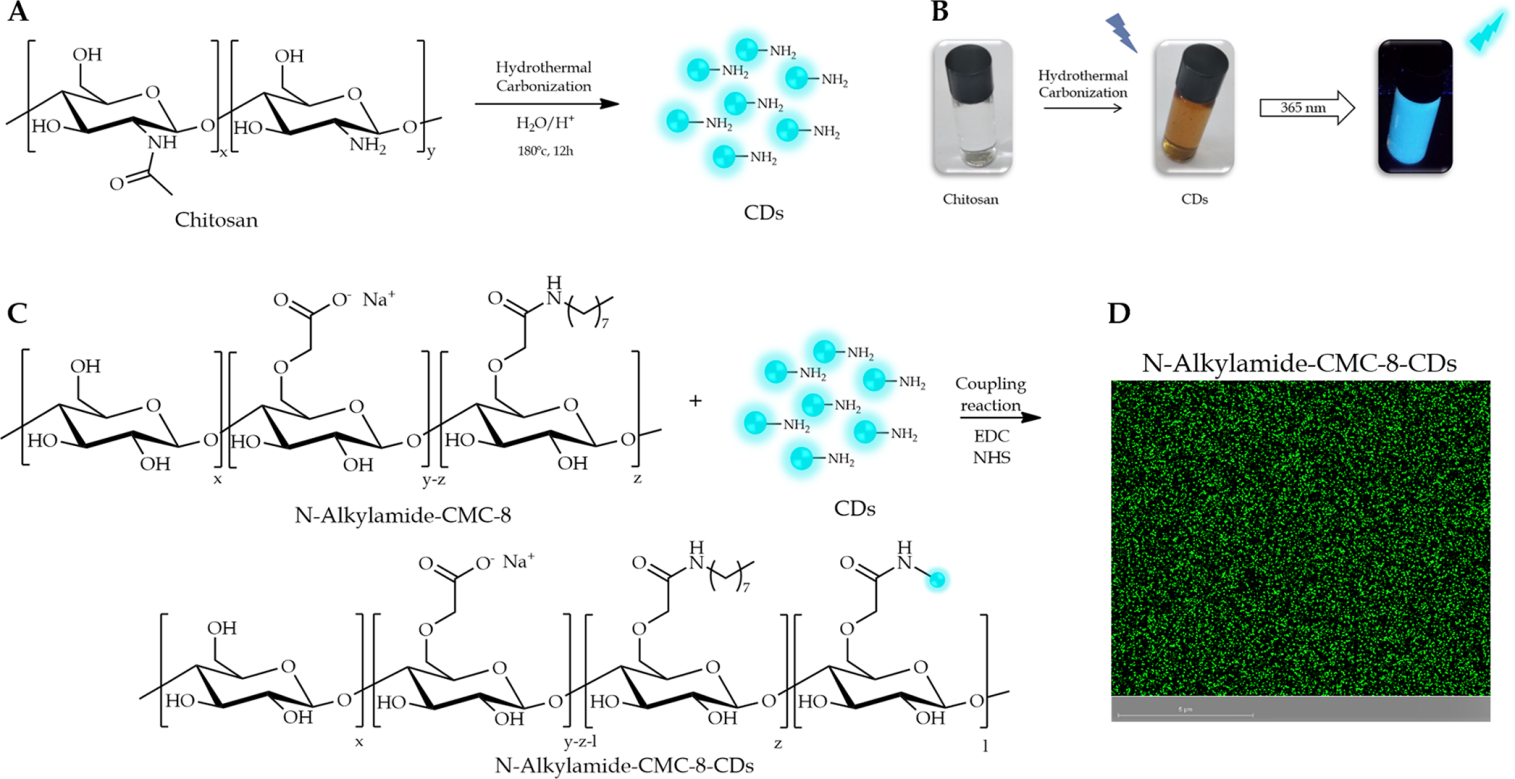
Synthesis of CDs (A) and the labeled CMC-8-CDs (C). Fluorescence presentation of chitosan CDs (B). CLSM images of labeled CMC-8-CDs. Scale bar is set to 5 μm (D).

Zinc uptake and translocation in plants were monitored by energy dispersive X-ray spectroscopy in conjunction with scanning electron microscope (EDS-SEM) and fluorescence microscope. In addition to monitoring zinc payload, we also aimed to view the *in planta* carrier, CMC-8. For this purpose, CMC-8 vehicles were labeled with fluorescent carbon dots (CD). The CD labeling method possesses numerous advantages in comparison to traditional fluorophores, because CDs do not affect solubility or activity of the labeled molecule.^47,48^ In this work, CDs were prepared *via* hydrothermal carbonization of polysaccharide chitosan^49^ and covalently attached to CMC-8 by amidation reaction resulting in photoluminescent CMC-8-CDs with a maximal emission intensity at 470 nm (Figures 6 and S5).

CMC-8 nanovehicles and the labeled nanovehicles (CMC-8-CDs) were characterized for their size, zinc encapsulation ability, and stability using TEM, SEM-EDS, and **ζ**-potential-DLS methods.

**Figure 7:**
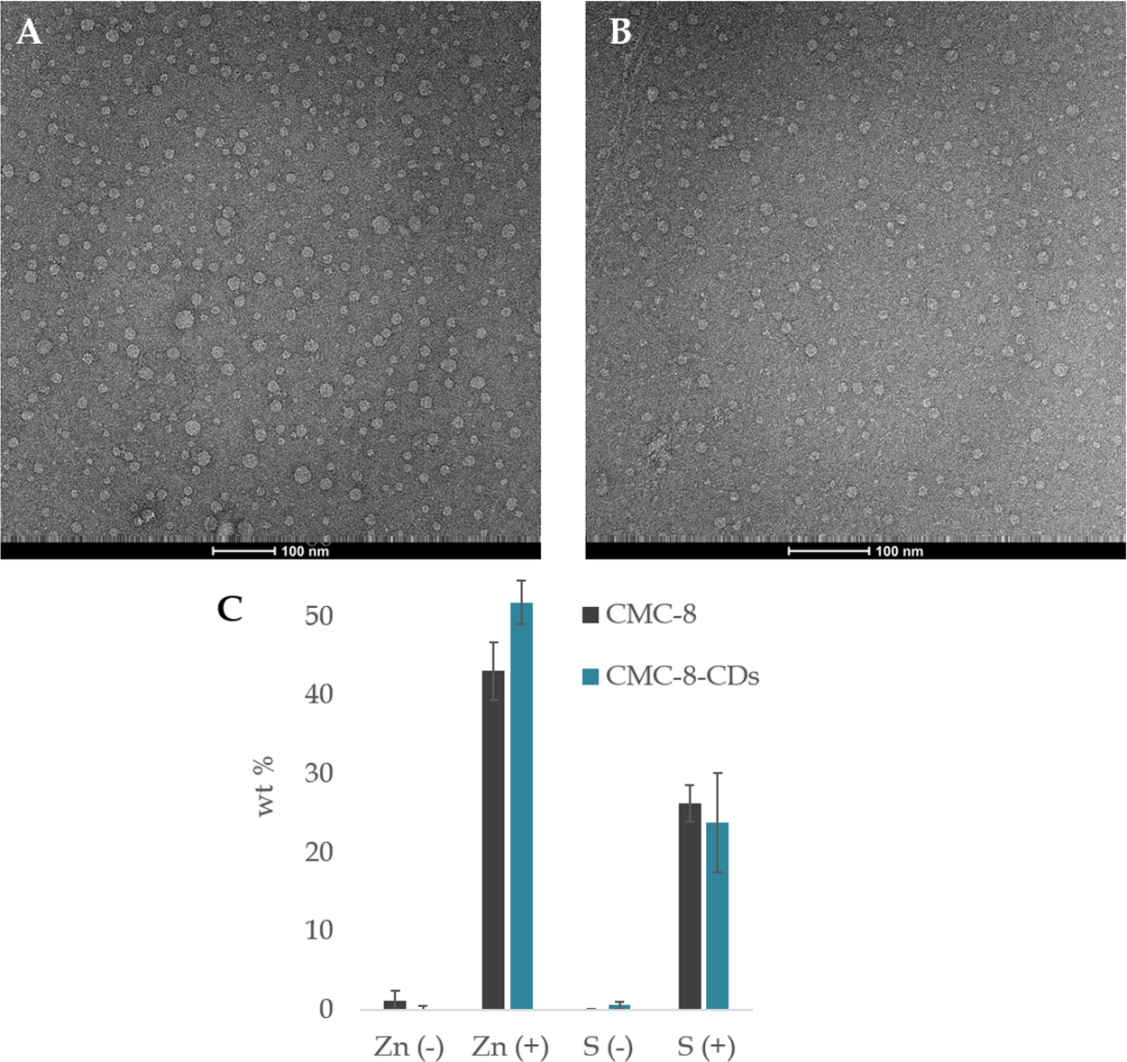
TEM images of (A) CMC-8 and (B) CMC-8-CDs in aqueous media in concentration of 15 mg/mL. Scale bar is set to 100 nm. (C) Zinc (Zn) and sulfur (S) weight percentage (wt %), calculated by EDS, in presence (+) and absence (−) of 500 ppm of ZnSO_4_ for CMC-8 (gray) and CMC-8-CDs (blue).

TEM images of CMC-8 and CMC-8-CDs present similar pattern, with numerous nanostructures of up to 15–40 nm size (the particle size range was calculated based on over 70 measurements of individual structures). SEM-EDS results indicated similar encapsulation ability for CMC-8 and the labeled CMC-8-CDs nanovehicles in the presence of 500 ppm ZnSO_4_ (Figure 7).

ζ-Potential measures aggregation resistance of colloidal dispersion; the higher the absolute ζ-potential values, the more stable a dispersion is. Because CMC-based structures are negatively charged, their ζ-potentials are negative. ζ-Potential values of CMC-8 and CMC-8-CDs showed similar, highly negative values of −54.4 and −55.4 mV, respectively, indicating the good stability of the prepared nanovehicles. Upon the addition of ZnSO_4_, ζ-potential values became less negative because of the encapsulation of positively charged ionic zinc, resulting in −23.3 and −25.4 mV for CMC-8+Zn and CMC-8-CDs+Zn, respectively (Table S1).

**Figure 8:**
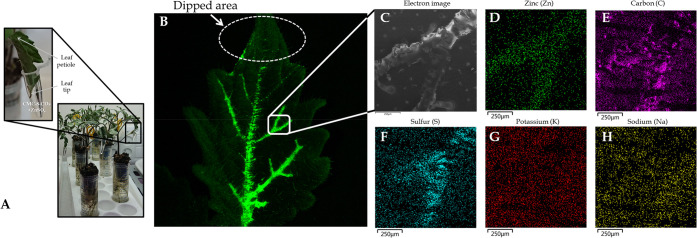
(A) Leaf dipping experience illustration. (B) Fluorescence stereoscope and (C–H) EDS images of tomato leaf dipped into CMC-8-CDs solution, after 24 h.

The tomato (*S. lycopersicum*) leaf’s tip was dipped into aqueous solutions of the labeled CMC-8-CDs loaded with ZnSO_4_, and movement of the nanovehicles was monitored by EDS-SEM and fluorescence stereoscope. The EDS-SEM shows the increased densities of carbon originated from the CMC-8 carrier; zinc and sulfur originated from the payload. The fluorescence stereoscope images were in correspondence with EDS-SEM, implying transportation of the loaded CMC-8 through the vascular system of the plant. These observations confirm the ability of CMC-8 vehicles to move against the water transport, from the leaf tip downward the leaf petiole (Figure 8).

Thus, CMC-8 nanovehicles show aptitude to transport ionic zinc through plant cuticle without an external stimulation. Despite the acquired hydrophobicity, CMC-8 has effectively encapsulated and delivered the highly hydrophilic payload to pepper and tomato plants upon foliar application. Notably, the unmodified CMC did not show any increase in the Zn content (Figure 5). In addition, the labeling experiments showed that nanovehicles enter the plant together with the loaded zinc. The size and amphiphilicity of the nanovehicles imply their possibility of crossing the cuticle by a cuticular or stomatal pathway and symplastic or apoplastic transport.^21^ All the mentioned possibilities can be a potential mechanism that still needs to be clarified.

These results present an interesting phenomenon, where introduction of hydrophobic substituents endow the polysaccharide with affinity toward the ionic microelement. In general, modification of delivery systems with hydrophobic substituents leads to an increase in their affinity toward hydrophobic payloads. Encapsulation of ionic microelements in the lipid systems such as liposomes has been done;^24^ however, to the best of our knowledge, an increase of the delivery system’s affinity toward a highly hydrophilic payload due to modification with hydrophobic substituents has never been reported.

## Conclusions

An amphiphilic polysaccharide-based delivery system, CMC-8, was synthesized, comprehensively characterized, and found to undergo spontaneous aggregation to form soft nanovehicles. The prepared nanovehicles effectively coordinated ionic zinc and demonstrated a capability to pass through the plant cuticle barrier without any external stimulation. Efficient uptake and diffusion of zinc were obtained upon foliar treatment of pepper and tomato plants. Chitosan-based fluorescent carbon dots were synthesized and covalently attached to CMC-8 nanovehicles allowing their *in planta* monitoring.

Three-dimensional structural modeling and conformational dynamics computational studies allowed us to understand the self-assembly mechanism and explain the observed phenomena, where the presence of hydrophobic substituents in CMC-8 enhances the ability to coordinate highly hydrophilic zinc cations.

The presented experimental and theoretical findings may contribute to the development of a green, feasible system for effective foliar delivery of bioactive agents to plants and may find additional biotechnological advances in diverse fields.

## Experimental Section

### Materials

Sodium carboxymethyl cellulose (Mw = 250 kDa; DS= 0.9), *n*-octylamine, and *n*-hydroxysuccinimide (NHS) were purchased from Acros Organics (Geel, Belgium). Acetic acid, pyrene, and ethylenediaminetetraacetic acid (EDTA) were purchased from Sigma-Aldrich (Steinheim, Germany). 1-(3-(Dimethylamino)propyl)-3-ethylcarbodiimide hydrochloride (EDC) was purchased from Alfa Aesar (Lancashire, United Kingdom). Dextran standards were purchased from PSS Polymer (Mainz, Germany). Zinc sulfate heptahydrate was purchased from Fisher chemicals (Dublin, Ireland). Chitosan (Mw = 890 kDa; DD ≥ 90%) was purchased from Glentham (Corsham, United Kingdom). Ethanol and ethanol absolute were purchased from Gadot-group (Netanya, Israel). Water (HPLC grade) was purchased from Bio Lab (Jerusalem, Israel). Deionized water (DW) was obtained by mechanically filtering them through a Treion TS1173 column. Deuterated solvent for NMR analysis (D_2_O) was purchased from Armar Chemicals (Döttingen, Switzerland). All regents and solvents were used without further purification.

### Methods

#### Preparation of Modified CMC

*N*-Alkylamidated CMC-8 was prepared by dissolving 0.58% w/w carboxymethyl cellulose (CMC) in 100 mL of DW at 60 °C. When the solution achieved homogeneity, the solution was cooled to room temperature, then 1.3 mmol of EDC and NHS were added. After 2.5 h, octyl amine was added at 1.3 mmol, and the solution was stirred overnight. According to the manufacturer’s specifications, the commercial CMC’s degree of substitution is DS = 0.9, and its carboxymethyl groups were calculated as (0.571 gr/270 g/mol)·0.9, where 0.571 gr is CMC’s mass, 270 is the molecular weight of glucose monomer, and 0.9 is the molar fraction of the monomer. Octyl amine, EDC, and NHS were thusly added at 0.7 equiv with respect to CMC’s carboxymethyl groups. CMC-8 was precipitated by adding 6 times the volume of ethanol compared to the reaction solution volume. It was then isolated *via* centrifugation (5000 rpm), washed 3 times with ethanol, and dried on a vacuum desiccator overnight.

#### Preparation of Chitosan CDs

The chitosan-based carbon dots (CS-CDs) were prepared based on a previous procedure.^49^ In brief, 2 g of chitosan was dissolved in 42 mL of 2% acetic acid aqueous solution. The solution was sealed into a Teflon equipped stainless steel autoclave for hydrothermal treatment at 180 °C. After 9 h, the autoclaved material was cooled naturally, followed by centrifugation at high speed (10 000 g-force) for 20 min at 4 °C in order to remove the less-fluorescent deposit. The strong fluorescent brown solution was lyophilized to produce a CS-CDs brown powder.

#### Preparation of CMC-8-CDs

CMC-8 (300 mg) was dissolved in DW at 60 °C. Once the solution achieved sufficient homogeneity, the solution was cooled to room temperature and 0.18 equiv of EDC and NHS were added. After 2.5 h, CS-CDs were added at 0.18 equiv, and the solution was stirred overnight by covering with aluminum foil. The solution was lyophilized to obtain a crude brown powder. The crude powder was washed 3 times with ethanol and dried overnight in a vacuum desiccator.

#### ATR FTIR

ATR-FTIR spectroscopy was conducted using a Thermo Scientific Nicolet iS5 FTIR spectrometer (United States). CMC and CMC-8 powders were subjected to 32 scans at a 0.5 cm^–1^ resolution between 500 and 4000 cm^–1^.

#### NMR

^1^H spectra were performed using Bruker Avance I and Avance III NMR 400 MHz spectrometers (United States). Chemical shifts are presented in parts per million (ppm). The solvent residual peak (H_2_O at 4.79 ppm) was used as calibration peak. All NMR samples were prepared using D_2_O as the solvent at 298 K. CMC peaks were found to be in accordance with previous publications.^50^

#### Degree of Substitution

The total organic carbon (TOC) and the total nitrogen (TN) were calculated by an elemental analyzer. Measurement of C and N concentrations was done using Shimadzu, ASTM D 8083 analyzer, United States (TOC-L with TNM), in water by high-temperature catalytic combustion and chemiluminescence detection. CMC and CMC-8 powders were dissolved in DW to give final concentration of 0.2 mg/mL.

The percentage CMC substitution of octyl amide was calculated by the following equation:
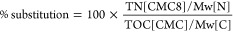


#### GPC

Molecular weights and polydispersity indices of the CMC and CMC-8 were calculated using gel permeation chromatography (GPC). Waters’ Alliance system e2695 separations module was used (Waters, United States), equipped with a refractive index detector, model blue 2414. The mobile phase used was HPLC grade water under isocratic elution for 30 min at a flow rate of 0.7 mL/min. The injection volume was 20 μL, and the temperature of both the detector and columns was 30 °C. Analyses were carried out using an Ultrahydrogel column, 1000 Å, 12 μm, 7.8 mm × 300 mm, 2–4000 kDa (Waters, United States). The molecular weights were calculated relative to standard dextran known molecular weight with *M*_n_ (the number-average molecular weight) range of 3 300–333 000 Da. All data provided by the GPC system were collected and analyzed with the Empower 3 personal dissolution software. CMC and CMC-8 powders were dissolved in the mobile phase to give a final concentration of 1 mg/mL. The solutions were filtered through a 0.22 μm nylon syringe filter.

#### TGA

Thermogravimetric analysis (TGA) was conducted using a PerkinElmer TGA 8000 instrument (TA Instruments, United States). Ceramic crucibles were loaded with 1–3 mg of each sample and heated from 25 to 800 °C with a heating rate of 10 °C/min under the flow of N_2_ (20 mL/min).

#### DSC

Differential scanning calorimeter (DSC) measurements were performed with a PerkinElmer DSC 6000 instrument (United States) calibrated by means of indium and zinc standards. Thermograms of each sample were obtained from the second heating run up to 440 °C, after the first run of heating up to 160 °C and cooling to 50 °C at a constant rate of 20 °C/min, under an N_2_ purge of 20 mL/min. Aluminum crucibles with pierced lids were loaded with 5–15 mg of each sample, and an empty aluminum crucible was used as the reference.

### Spectrofluorometry

#### Critical Aggregation Concentration Measurements

The fluorescent emission spectrum of pyrene (fluorescent probe) comprises vibronic peaks that show a strong dependency on the polarity of solvent.^51,52^ The ratio between two specific peaks (*i.e.*, *I*_3_ ≈ 383 nm and *I*_1_ ≈ 373 nm) in pyrene’s spectrum was used as a quantitative measurement for its microenvironment’s polarity. Any change in the surrounding polarity, such as when pyrene is encapsulated from an aqueous environment by a hydrophobic-cored aggregate, is expressed by this ratio’s value.^53^ Pyrene’s highly hydrophobic nature and low water solubility (2–3 μM) ensure its preference to be present in hydrophobic over hydrophilic environment. These characteristics make pyrene an ideal fluorescent probe for analyzing a substance’s critical aggregate concentration. Diluent solution was prepared by adding 25 μL of pyrene stock solution (0.49 mg/mL in Ethanol) into 50 mL of DW, to give a final concentration of 1.2 μM. A 15 mg/mL of CMC-8 solution was dissolved in the above diluent, and the mixture was stirred overnight. This solution was repeatedly diluted by a factor of 2 with the above diluent. Then, 150 μL of each solution was loaded onto a 96 well plate. The fluorescence emission intensity from each well was scanned using a Synergy HTX multimode reader device (BioTek Instruments Inc., Winooski, United States). The excitation wavelength for pyrene was 340 nm, and the emission band recorded was 360–400 nm, at increments of 1 nm. All samples were made in triplicate. CAC values were calculated as the intersection between two linear lines depicting aggregate formation dependent on concentration in solution.^53^

#### CDs Fluorescence Measurements

CDs fluorescence spectra of CS-CDs (4 mg/mL) samples were measured in a standard 1 cm quartz cell using a computer-controlled Shimadzu, RF-5301PC spectrofluorometer equipped with a 150 W xenon lamp (Ushio Inc., Japan). The excitation wavelength range for CS-CDs was 330–550 nm with a slit width of 3 nm, and the emission band recorded was (λ_ex_ + 20) – 700 nm with a slit width of 3 nm, at increments of 0.5 nm.

#### Confocal Microscopy

Laser scanning confocal microscopy (CLSM) imaging was performed using a Leica SP8 laser scanning microscope (Leica, Wetzlar, Germany), equipped with an OPSL 488 nm laser, HC PL APO CS2 63×/1.20 water objective (Leica, Wetzlar, Germany), and Leica Application Suite X software (LASX, Leica, Wetzlar, Germany). CMC-8-CDs (16.4 mg/mL) were excited at 488 nm, and the emission signal was detected with HyD (hybrid) detector in a range of 500–550 nm.

#### TEM

Samples (3 μL drop) of CMC-8 and CMC-8-CDs aqueous solution (15 mg/mL) and the same solutions with addition of 500 ppm of zinc (CMC-8+Zn and CMC-8-CDs+Zn) were placed on glow-discharged carbon-coated 300 mesh copper TEM grids (Ted Pella, Inc.). After blotting, the samples were negatively stained with 2% aqueous solution of uranyl acetate for 40 s and air-dried. The samples were examined by a FEI Tecnai 12 G2 TWIN TEM operated at 120 kV. Images were recorded using a 4k × 4k FEI Eagle CCD camera (ThermoFisher scientific, United States).

#### ζ-Potential

Electrokinetic properties (ζ-potential) were determined by a Zetasizer (3000 HSa, Malvern Instruments Ltd., U.K.). ζ-Potential values of CMCs were measured at 4 mg/mL in DW. The measurements were done in triplicate, and the obtained values represent means ± standard error.

#### Dynamic Light Scattering (DLS)

All measurements were recorded on a Nano ZS ZEN3500 Malvern light-scattering photometer equipped with a 50 mW laser at an operating wavelength of 532 nm. All measurements were conducted at 23 °C (refractive index, 1.330; viscosity, 0.890 cP for water) with an angle detection (θ) of 173°. CMC samples were prepared by dissolving 4 mg/mL in DW. Each sample was measured three times, and the average Dh and polydispersity (PDI) values were calculated.

#### Zinc Encapsulation Measurements

*In vitro* evaluation of the ability of CMC-8 and CMC-8-CDs interaction with ZnSO_4_. Drops (3 μL) of the different formulations at 2 mg/mL and 500 mg/L Zn (ZnSO_4_) were put on a carbon tape and allowed to dry at room temperature. Six drops were evaluated for each formulation and cargo combination (with and without ZnSO_4_). Carbon tapes were mounted on a stub and scanned under SEM (VEGA3, Tescan, Czech Republic) coupled with an EDS detector (model X-act, Oxford Instruments, U.K.) according to Tan *et al.*(64) Zn, S, Na, Mg, and Cl distribution and content at different locations on the leaf surface were detected with the SEM-EDS. The distributions and content of the above elements are presented as mass percentage (mass %) of the total weight of all analyzed elements. SEM was operated with acceleration potential of 20 kV at a working distance of 15 mm and highest beam intensity (18 units in our specific SEM) and a spot size of 500 nm, and each scan was for 3 min. To avoid missing results between Na and Zn due to overlapping electron energies, Zn was detected only by its Kα energy.

### Computational Methodology

#### Statistical Properties and 3D Models of CMC Derivatives

The most detailed description of the statistical properties of CMC at different DS is given elsewhere.^54^ This experimentally derived/validated data enables the correct modeling of *N*-alkyl amide CMC derivative, CMC-8. According to this study, for a typical 1000-mer CMC chain with DS ≈ 0.9, we could expect 360–370, 160–170, and 350–360 substitutions at positions 2, 3, and 6, respectively (Table 2 in Heinze and Pfeiffer).^54^ Further, the mole fractions of unsubstituted, mono-, di-, and trisubstituted glucose monomers in this CMC 1000-monomer chain are 330–340, 440–450, 190–200, and 20–30, respectively (Table 3 in Heinze and Pfeiffer).^54^ These two distributions given for CMC with DS ≈ 0.9^54^ were used as a template for the structural modeling of *N*-octyl-amidated CMC (CMC-8) polymer chains.

The CMC-8 macromolecules were modeled as follows. In a cellulose chain composed of 1000 d-glucose units, 900 hydroxyls were randomly substituted by carboxymethyl moieties (corresponding to DS 0.9). We have generated 100 random CMC polymer chains. The average composition of mono-, di, and trisubstituted glucose monomers agreed with the statistics experimentally observed in CMC with DS 0.9.

Further, structures of CMC-8 polymers were modeled by random *N*-octyl-amidation of CMC carboxy groups according to our experimental DS value of 22%, determined for *N*-octyl-amide CMC derivative. For example, 200 carboxy groups were randomly replaced by *N*-octyl-amide moieties/groups.

#### Molecular Modeling, Conformational Analysis, and Electrostatics

In the computational methodology applied to the CMC-based polyelectrolyte systems, the experimental and numerical data on the distribution of the carboxymethyl- (CM) and the *N*-octyl amide moieties in CMC chains were used, as described above.

Further, the methodology comprised the following steps:

(1) *In silico* generation of initial 3D structures of the CMC-based polymer molecules with the realistic distribution of CM and *N*-octyl amide CM groups in polymer chains was done to mimic *in vitro* compositions. Random structures with polymer chain lengths 100 and 1000 were generated. For CM groups, the DS ≈ 0.9 in cellulose chains was used. The 22% (0.22) DS of the CM groups by *N*-octyl amide groups was taken as explained above. These *in silico* substitutions in the cellulose chains were generated using our python program SubRandoMol.py and the Indigo library/modules.^55^ The cellulose chains were built using CarbBuilder program.^56^

(2) Conformational sampling and the analysis of semirigid CMC and CMC-8 chains by using molecular dynamics (MD): Multiple 10–100 ns MD trajectories were generated for single 100 mer CMC and CMC-8 chains by using the MD protocols.^57,58^ Different CMC and CMC-8 single chains were placed into the 53 × 3 × 3 nm^3^ box filled by ∼14 900 solvent (water) molecules. Total charges of CMC (−90*e*) and CMC-8 (−70*e*) molecules were neutralized by 90 and 70 sodium (Na+) cations, respectively. Molecular topologies based on amber99SB and the carbohydrate GLYCAM06 force fields,^59^ were prepared for GROMACS MD simulations using AmberTools and acpype programs.^60^ For each trajectory, snapshots were collected, and the persistence length of CMC and CMC-8 chains and conformational flexibility of CM groups and *N*-octyl-amide-CMC chains were analyzed.

(3) Continuum electrostatics model of the CMC-based polymers: In our electrostatics calculations, we assumed that CMC and *N*-octyl-amide CMC polyelectrolyte chains are placed into a high-dielectric solvent (water, with the dielectric constant ∼80), and surrounded by mobile Na^+^/Cl^–^ ionic cloud. The ∼100 mM (0.1 M) salt concentration was used. Distribution of the electrostatic potential around CMC and CMC-8 polymer molecules was calculated using the nonlinear Poisson–Boltzmann equation solver (NLPB) implemented in the APBS program.^61^ Computational details are given in Tworowski *et al.*(62)

(4) Self-aggregation of CMC-8 polymer molecules: Molecular models of self-assembled aggregates were created and optimized by using PackMol program.^63^ Random mixtures of 2700 conformers of CMC-8 molecules with a chain length of 100 were placed into a 600 Å cubic box. These parameters correspond to a CMC bulk density of ∼0.6 g/cm^3^.

### Plant Model Experience

#### Model Experience- Zn Staining

Pepper leaf and leaf petiole were chosen as “hard” petiole that allow easy hand cross sectioning and allow for Zn staining analysis; tomato plant leaf was chosen because of its leaflets’ pointy shape which allows easy operation of the dipping experiments.

The histochemical techniques of zinc (Zn) visualization are based on the formation of the green-fluorescent complex with Zinpyr-1 (C_46_H_36_Cl_2_N_6_O_5_). Ten 3 μL drops were applied using a pipet to the bottom part of the pepper leaf (*C. annuum*), just above the petiole (9 leaves per treatment, Figure S4). Treated plants were kept at room temperature for 24 h; hand-cut cross sections of the petiole were done using a razor blade, and sections were put in DW. Sections were transferred to a Zinpyr-1 solution (10 μM) and kept in the dark for 1 h at room temperature. After Zinpyr-1 incubation, sections were washed in DW and observed using a fluorescence stereoscope. The fluorescent images were captured with Nikon SMZ25 zoom stereoscopes. Zinpyr-1 signal was excited by a 500 nm filter, where the emission was collected by a 535 nm filter (YFP). Petiole vascular was detected using excitation by a 405 filter, where the emission was collected by a 460 nm filter (BFP). Images were captured with a color camera (DS-Ri2, Nikon) operated by NIS Elements BR software (Nikon). Zinpyr-1 quantification was performed on the stereoscope images of petiole cross sections using the software NIS Elements BR software. For each analysis, nine petiole cross sections were measured. Zn concentration was adjusted to 500 ppm for all the samples (EDTA-Zn, CMC-Zn, and CMC-8-Zn) in addition to H_2_O; MgSO_4_ (500 ppm) and CMC-8 were used as negative controls; the CMC and CMC-8 concentrations were maintained at 4 mg/mL.

#### Carbon Dots

Tomato leaves (*S. lycopersicum*) were used for *in vivo* assessment of CMC-8-CDs using a leaf-dipping methodology (Figure 8A). Leaflets were immersed in an Eppendorf tube containing CMC-8 or CMC-8-CDs solutions at 4 and 500 mg/L Zn (ZnSO_4_) for 24 h. Leaves were placed on a microscope stub and scanned in the scanning electron microscopy instrument (SEM; VEGA3, Tescan, Czech Republic) coupled with an energy-dispersive X-ray spectroscopy detector (EDS; model X-act, Oxford Instruments, U.K.).^64^ Zn, C, S, K, and Na distribution and content at different locations on the leaf surface were detected with the SEM-EDS. The distributions and content of the above elements are presented as mass percentage (mass %) of the total weight of all analyzed elements. The SEM was operated with an acceleration potential of 20 kV at a working distance of 15 mm, highest beam intensity (18 units in our specific SEM), and a spot size of 500 nm, and each scan was for 3 min. To avoid missing results between Na and Zn because of overlapping electron energies, Zn was detected only by its Kα energy. Before sampling for the SEM-EDS, the treated leaflet was observed under a fluorescence stereoscope (SMZ25, Nikon) using YFP filter (excitation wavelength, 500 nm; emission wavelength, 535 nm), and the image was taken using DS Ri2 color camera (Nikon). Stereoscope operation and image analysis was conducted using Nis Elements Br (Nikon). The zinc content in the tomato leaves treated with the regular CMC-8 and the labeled CMC-8-CDs calculated by SEM-EDS resulted in similar values, which confirm that CDs do not affect the delivery system’s performance.

## Supporting Information Available

ζ-potential, DLS, and polydispersity values; composition of 27 distinct monomers for CMC and CMC-8 macromolecule; pepper leaf application image; ATR-FTIR spectra of chitosan and chitosan carbon dots (CS-CDs); fluorescence emission spectra of CS-CDs (PDF)
