# Supplementary material for: Stimuli-Free Transcuticular Delivery of Zn Microelement Using Biopolymeric Nanovehicles: Experimental, Theoretical, and In Planta Studies
Source: ACS Nano. 2021 Nov 24;15(12):19446–56. doi: 10.1021/acsnano.1c06161 (PMC8900126; doi:10.1021/acsnano.1c06161)
Supplement: Supplementary file 1 — nn1c06161_si_001.pdf [file nn1c06161_si_001.pdf]

-Supporting Information-

Stimuli-Free Transcuticular Delivery of Zn Microelement Using Biopolymeric Nanovehicles: Experimental, Theoretical and *in Planta* Studies

Yael Cohen,[2,1] Hagai Yasuor,[3] Dmitry Tworowski,[4] Elazar Fallik,[1] and Elena Poverenov[*1]

[1] Agro-Nanotechnology and Advanced Materials Center, Institute of Postharvest and Food Sciences, Agriculture Research Organization, The Volcani Center, Rishon LeZion 7505101, Israel

[2] Institute of Biochemistry, Food science and Nutrition, Faculty of Agriculture, Food and Environment, The Hebrew University of Jerusalem, Rehovot 76100, Israel

[3] Department of Vegetables and Field Crops, Agriculture Research Organization, Gilat Center, M. P. Negev, 85280, Israel

[4] Department of Structural Biology, Weizmann Institute of Science, 76100 Rehovot, Israel

[*] Corresponding author E-mail: elenap@volcani.agri.gov.il, Tel: 972-39683354. Agricultural Research Organization, 68 HaMaccabim Road, P.O.B 15159 Rishon LeZion 7505101, Israel.

Table S1 ζ-potential and DLS and polydispersity values for CMC-8, CMC-8+Zn, CMC-8-CDs, CMC-8-CDs+Zn.

	ζ-potential [mV]	Dh [nm]	PDI
CMC-8	-55.4±2.53	53.48±18.40	0.541
CMC-8+Zn	-23.3±1.21	13.47±1.51	0.693
CMC-8-CDs	-54.4±1.87	46.78±10.70	0.675
CMC-8-CDs+Zn	-25.4±2.55	9.26±1.60	0.685
CDs	-9.64±3.73	1.46±0.77	0.91

The composition of the CMC and *N*-alkyl-amide CMC-8 is extremely complex. The CMC polymer chain is a copolymer of 8 different monomers depending on the substitutions at the positions 2,3, and 6 in the glucose moiety (Figure S1). These monomers are represented by unsubstituted glucose (u), three mono-substituted (2, 3, or 6) derivatives (m), three di-substituted glucose monomers (d), and the monomer with all three hydroxy groups modified by carboxymethyl moiety (Figure S1). The partial modification of the CMC monomers by *N*-octyl groups (22 %) brings the macromolecule to a higher level of complexity. The substitutions in mono- and di- CMC moieties give 12 different *N*-alkyl monomers (Figure S2), and all possible modifications in the 2,3,6-CMC moiety produce another group of 7 new monomers (Figure S3).

Therefore, an *N*-alkyl-amide-CMC macromolecule is a composition of 27 distinct monomers randomly distributed along a polymer chain. For the initial DS 0.9 followed by the 22 % *N*-alkyl-amidation, the *N*-octyl-amide-CMC (CMC-8) polymer chain of a 250 kDa macromolecule is estimated to be approximately 1000 monomers length. We generated a library of random 1000-mer sequences of 27 monomers (Figures S1-S3) corresponding to the composition of the CMC-8 with DS 0.9; numerical data.[1]

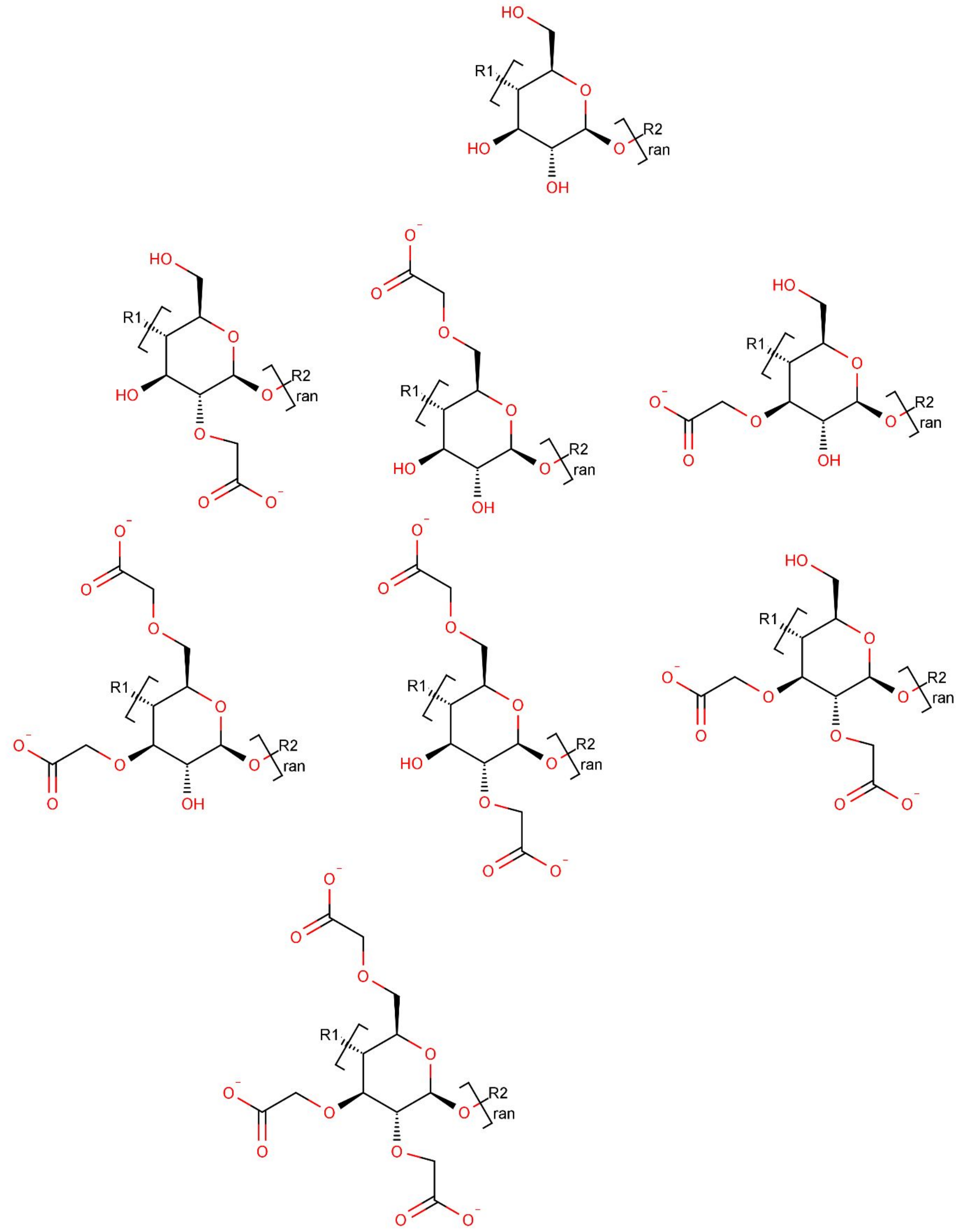

Figure S1 Molecular composition of 8 distinct monomers for CMC.

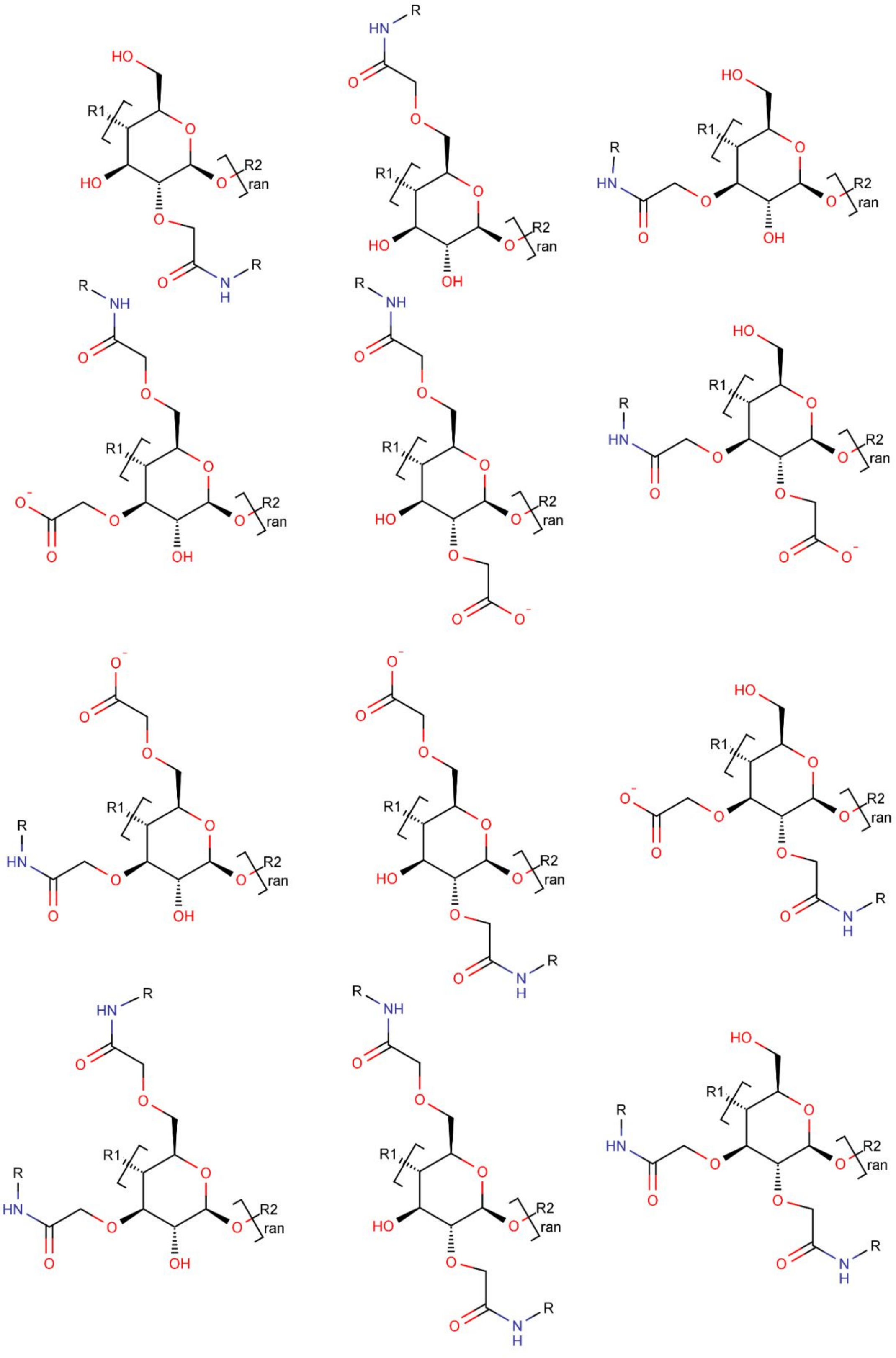

Figure S2 Molecular composition of 12 distinct monomers for CMC-8.

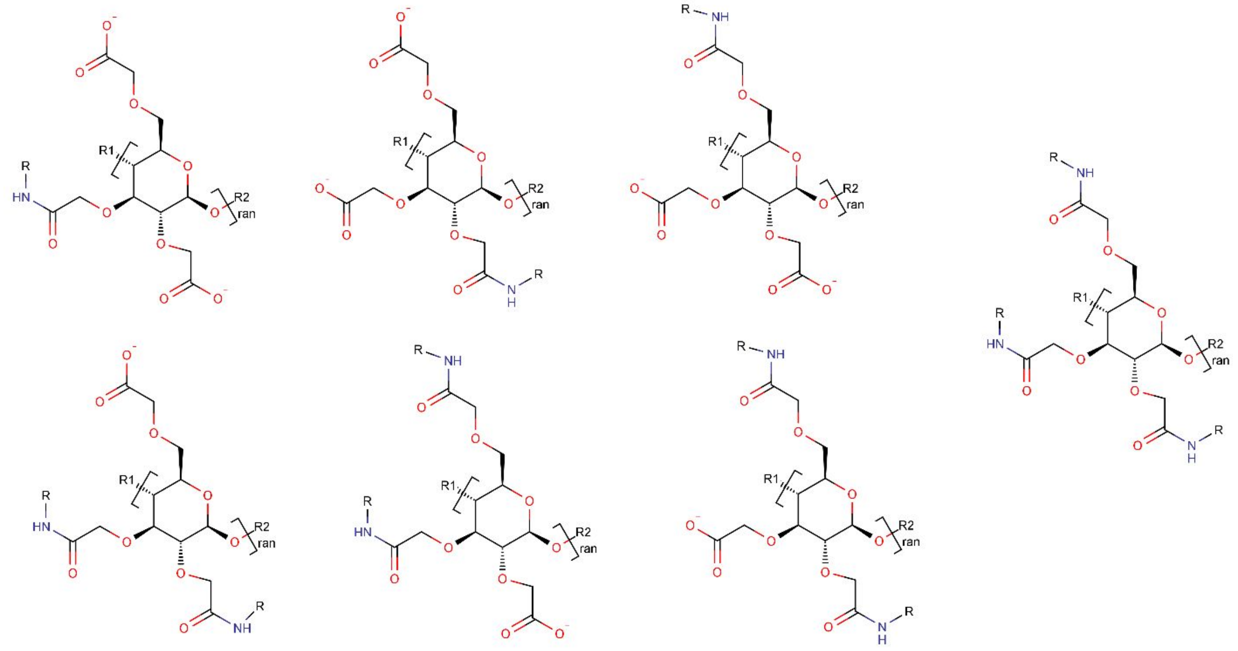

Figure S3 Additional molecular composition of 7 distinct monomers for CMC-8, by modifications in the 2,3,6-CMC moiety.

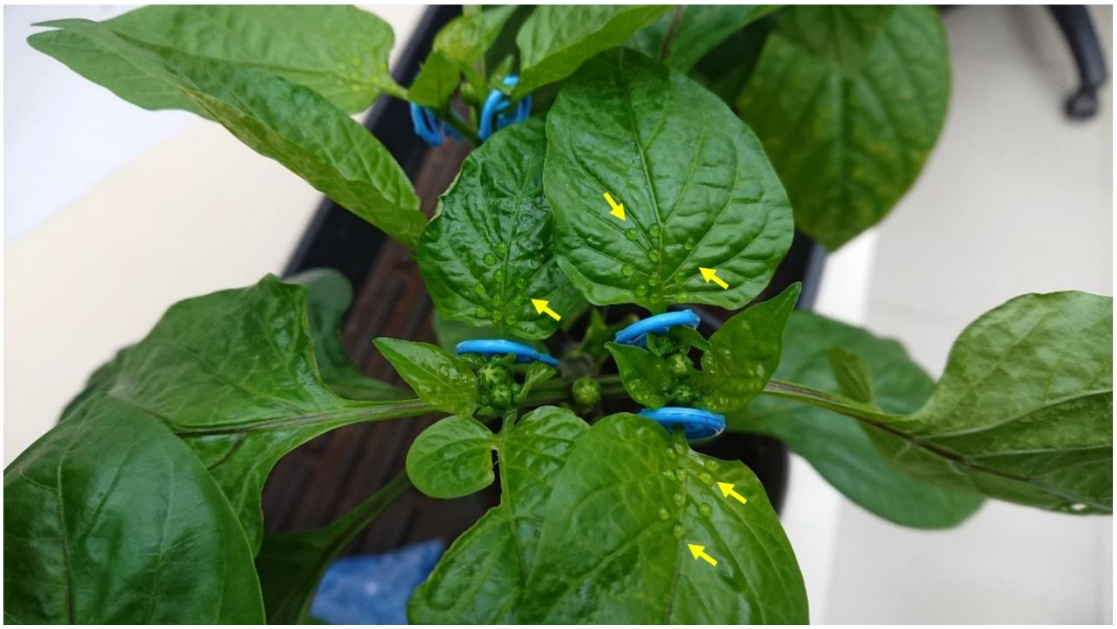

Figure S4 Pepper leaf application image.

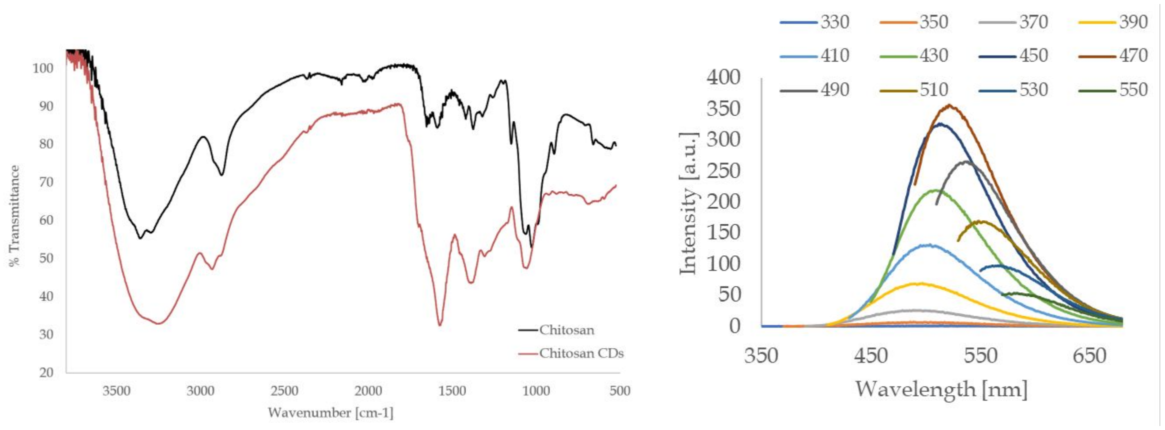

Figure S5 (left) ATR-FTIR spectra of chitosan and chitosan carbon dots (CS-CDs). (right) Fluorescence emission spectra of CS-CDs.

References

(1) Heinze, T.; Pfeiffer, K. Studies on the Synthesis and Characterization of Carboxymethylcellulose. *Die Angew. Makromol. Chemie* **1999**, *266* (1), 37–45. https://doi.org/10.1002/(SICI)1522-9505(19990501)266:1<37::AID-APMC37>3.0.CO;2-Z.
